# Swimming pool exposure is associated with autonomic changes and increased airway reactivity to a beta-2 agonist in school aged children: A cross-sectional survey

**DOI:** 10.1371/journal.pone.0193848

**Published:** 2018-03-12

**Authors:** João Cavaleiro Rufo, Inês Paciência, Diana Silva, Carla Martins, Joana Madureira, Eduardo de Oliveira Fernandes, Patrícia Padrão, Pedro Moreira, Luís Delgado, André Moreira

**Affiliations:** 1 Basic and Clinical Immunology, Department of Pathology, Faculty of Medicine, University of Porto & Immunoalergology Department S. João Hospital Centre, Porto, Portugal; 2 Energy and Built Environment Group, Institute of Science and Innovation in Mechanical and Industrial Engineering, Porto, Portugal; 3 EPIUnit—Instituto de Saúde Pública, Universidade do Porto, Rua das Taipas, n° 135, Porto, Portugal; 4 Faculty of Nutrition and Food Sciences of the University of Porto, Porto, Portugal; Universite de Bretagne Occidentale, FRANCE

## Abstract

**Background:**

Endurance swimming exercises coupled to disinfection by-products exposure has been associated with increased airways dysfunction and neurogenic inflammation in elite swimmers. However, the impact of swimming pool exposure at a recreational level on autonomic activity has never been explored. Therefore, this study aimed to investigate how swimming pool attendance is influencing lung and autonomic function in school-aged children.

**Methods:**

A total of 858 children enrolled a cross sectional survey. Spirometry and airway reversibility to beta-2 agonist, skin-prick-tests and exhaled nitric oxide measurements were performed. Pupillometry was used to evaluate autonomic nervous function. Children were classified as current swimmers (CS), past swimmers (PS) and non-swimmers (NS), according to the amount of swimming practice.

**Results:**

Current swimmers group had significantly lower maximum and average pupil constriction velocities when compared to both PS and NS groups (3.8 and 5.1 vs 3.9 and 5.3 vs 4.0 and 5.4 mm/s, *p* = 0.03 and *p* = 0.01, respectively). Moreover, affinity to the beta-2 agonist and levels of exhaled nitric oxide were significantly higher in CS when compared to NS (70 vs 60 mL and 12 vs 10 ppb, *p*<0.01 and *p* = 0.03, respectively). A non-significant trend for a higher risk of asthma, atopic eczema and allergic rhinitis was found with more years of swimming practice, particularly in atopic individuals (β = 1.12, 1.40 and 1.31, respectively). After case-case analysis, it was possible to observe that results were not influenced by the inclusion of individuals with asthma.

**Conclusions:**

Concluding, swimming pool attendance appears to be associated with autonomic changes and increased baseline airway smooth muscle constriction even in children without asthma.

## Introduction

Swimming is not only one of the most practiced sports worldwide, but is frequently recommended by physicians, due to the multiple associated benefits. These recommendations often target patients with allergic and respiratory diseases since it is believed that health benefits of indoor swimming significantly surpass the risks associated with the practice, such as drowning or infections [1].

However, the endurance swimming exercises coupled to disinfection by-products exposure has been shown responsible for increased airways dysfunction in elite swimmers [2–7]. Moreover, airways dysfunction, such as airway hyperresponsiveness and bronchoconstriction, has been associated with parasympathetic dysautonomia in athletes, including swimmers [8–10]. Shortly, airways are innervated by postganglionic parasympathetic-cholinergic nerves which, when activated, are capable of reducing the lumen of small bronchi and bronchioles, significantly increasing airways resistance. It is therefore comprehensible that if exogenous stimuli may influence the parasympathetic tonus, they may also indirectly affect airway smooth muscle constriction [11].

Swimming pool exposure is known to be responsible for airway dysfunction in swimmers due to chlorine-based disinfection by-products [5, 12, 13]. These products may be ingested, inhaled or absorbed via skin during the swimming practice, thus resulting in airways epithelium damage and eventually airway hyperresponsiveness [4, 14]. This has been emphasized by several studies published in the last decade, showing an association between swimming pool attendance and a higher risk of asthma in children [15–17], which has lead the scientific community to question the beliefs regarding swimming practice benefits in children [18]. Nevertheless, endurance exercises and extensive training volume are frequently held responsible for the aforementioned autonomic nervous function changes, somehow disregarding the possible influence of the indoor swimming pool environment on the parasympathetic tonus. In fact, environmental pollutants are known to promote a higher expression of transient receptor potential vanilloid 1 [19–21], which is the centre of almost all neuronal inflammatory signalling pathways [22]. However, the impact of swimming pool exposure on autonomic activity has never been explored. It is therefore possible that exposure in swimming pools is also associated with dysautonomia in swimmers, independently of the training volume.

To further explore this hypothesis, the present study aimed to investigate how swimming pool attendance may relate to airway constriction and dysautonomia in school-aged children.

## Methods

### Participants and study design

A cross-sectional survey of children attending the 3rd and 4th grades from 20 primary schools in Porto, Portugal, was conducted from January 2014 to March 2015. The study was authorized by the Ethics Committee for Health of S. João Hospital Centre and by the schools’ Directive Councils. Parents and legal guardians of 1602 children attending the participating schools were contacted and received written information concerning the project. A written consent was retrieved for 916 children (57.2% participation rate), but only 858 (aged 7 to 12 years old) completed the clinical assessment as the remaining 58 children refused to perform some of the clinical tests despite the legal guardians’ consent. Height, weight, lung function (spirometry with bronchodilation) and exhaled nitric oxide (NO) levels were measured in participating children. Skin-prick-tests (SPT) and pupillometry were also performed on the participants by a trained professional. A standardized ISAAC-based self-reported questionnaire focused on their child’s respiratory and allergic symptoms, as well as on swimming practice, was filled by the parents.

### Physiological assessments

Children’s lung function and airway reversibility were assessed according to the ATS/ERS guidelines [23]. Lung function data was retrieved before and 15 minutes after bronchodilation, which was stimulated by administering an inhaled beta-2 agonist (400 μg of inhaled salbutamol).

Eosinophilic airway inflammation was assessed by measuring exhaled NO levels using the NObreath (Bedfont Scientific Ltd. UK). The results were expressed as parts per billion (ppb) and stratified according to the official ATS guidelines for children [24].

Allergic sensitization was evaluated by SPT on their forearm using a QuickTest^TM^ applicator and extracts of *Dermatophagoides pteronyssinus*, weed pollen mix, grass pollen mix, cat dander, dog dander and *Alternaria alternata*, negative control (extracts dilutant), and a positive control (histamine at 10mg/mL), all belonging to the same batches (Hall Allergy, Netherlands). Results were read 20 minutes afterwards. If children were on antihistamines or topical corticosteroids on the skin within the previous 7 days, SPTs were postponed.

Regarding pupillometry, the participants spent 15 minutes in a semi-dark and quiet room to allow their eyes to adjust to the low lighting levels before measurement. Pupillary measurements were taken with the portable infrared PLR-200™ Pupillometer (NeurOptics Inc, CA, USA). The complete pupillometry methodology has been thoroughly described in previous publications [8, 25]. Shortly, the pupil constriction response to a light stimulus represents parasympathetic activity, and the dilatation represents sympathetic activity. The following parameters were recorded: percentage of pupil constriction (CON); average and the maximum constriction velocities (ACV and MCV, respectively); minimum and maximum pupil diameter; average dilation velocity (ADV); and the time in seconds at 75% recovery of pupil size (T75). Since there was no side-to-side difference in pupil responses, all pupillary data reported in the results was obtained from the right eye, in a similar approach to Muppidi *et al*. [26]. If a valid measurement was not obtainable, measurements from the left eye were used instead.

### Definition of clinical and exposure outcomes

To improve observational power on asthma prevalence, four different operational asthma definitions were adopted: i) *Clinical criteria*–at least a 12% and over 200mL increase in FEV_1_ after bronchodilation and/or self-report of asthma diagnosed by a physician with reported symptoms (wheezing, dyspnoea or dry cough) occurring in the past 12 months; ii) *Functional criteria*–at least a 12% and over 200mL increase in FEV_1_ after bronchodilation; iii) *Treated asthma*–self-report of asthma diagnosed by a physician and currently under inhaled corticosteroid treatment; and iv) *Ever asthma*–self-report of asthma diagnosed by a physician.

Allergic sensitization was defined by a positive SPT to at least one of the tested allergens (wheal > 3mm) coupled to a positive histamine response (wheal > 3mm) and no positivity in the negative control (wheal < 3mm) [27].

Atopic eczema was defined as a positive answer to the question “Did your child ever had itchy skin alterations that appeared and disappeared for at least 6 months, during the past 12 months?” followed by a positive answer to “Did these skin alterations ever affected elbow and knee joints, ankles, between thighs, or around the neck, ears or eyes?”, based on the UK Working Party diagnostic criteria for the definition on atopic eczema [28]. Otitis definition was based on a positive answer to the question “Did your child had ear pain or otitis in the last 12 months not associated with a cold or a flu?”, while allergic rhinitis was defined as a positive answer to the question “Did your child suffered from recurrent sneezing, runny nose or nasal congestion, in the past 12 months, not associated with a cold or a flue?”.

**Fig 1:**
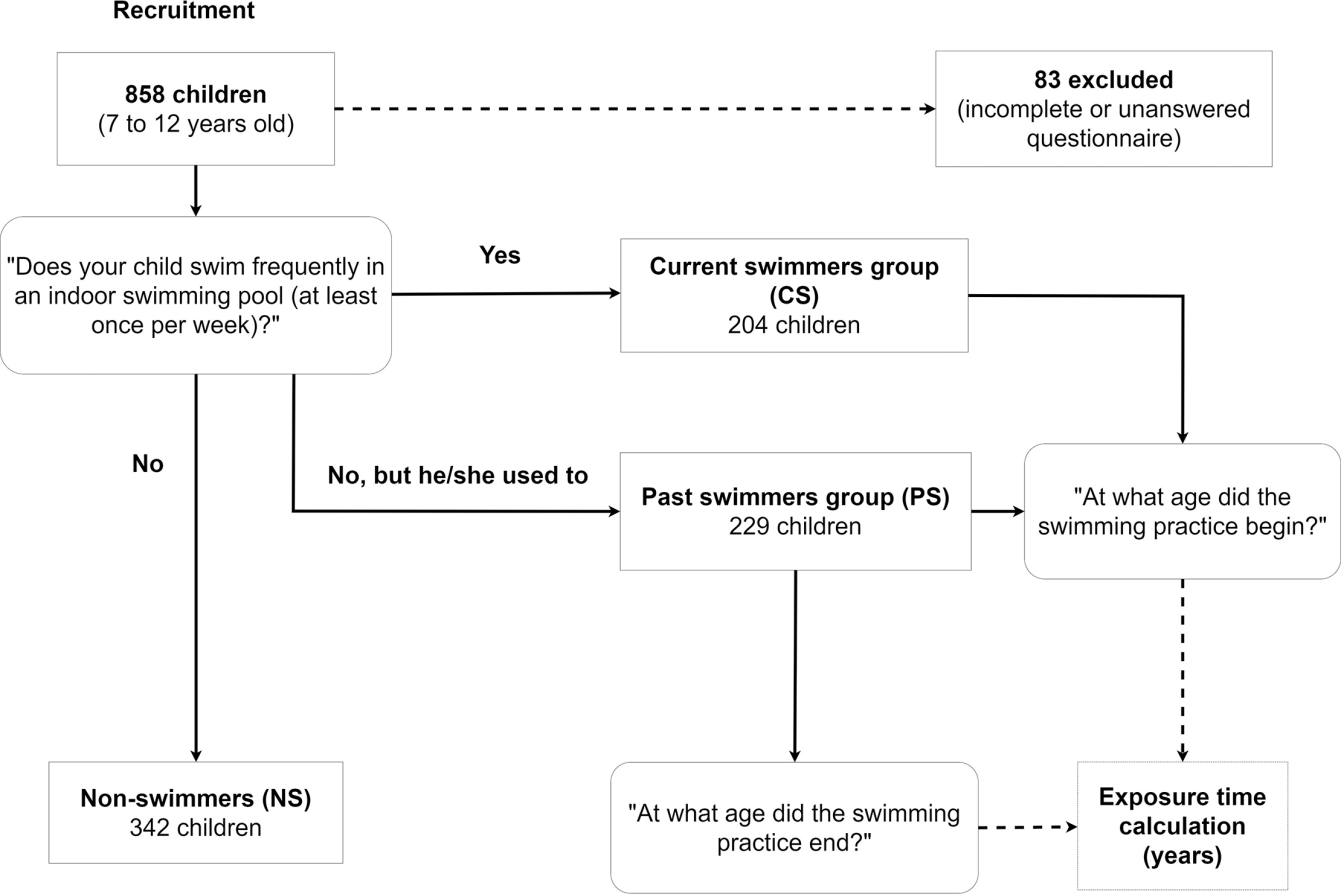
Flow chart of the recruited participants. Round-edged boxes represent the questions that allowed classification of participants into three different groups according to the respective answers: current, past and non-swimmers.

Through questionnaires, subjects were defined as *current swimmers* (CS) if parents answered “Yes” to the question: “Does your child swim frequently in an indoor swimming pool (at least once per week)?”; if they answered “No, but he/she used to”, participants were classified as *past swimmers* (PS); otherwise, if they answered “No”, they were regarded as *non-swimmers* (NS). For the CS and PS groups, cumulative swimming pool exposure, expressed in years, was calculated. Due to incomplete or unanswered questionnaires, 83 participants were excluded from the study. Therefore, a total of 205, 228 and 342 children were classified as CS, PS and NS, respectively. The question “Does your child partake in any sport activity outside of normal school-period, at least once per week?” was used to scrutinize if non or past swimmers practiced any other type of sport, in order to exclude any conceivable bias associated with sedentarism or training volume. Fig 1 illustrates the recruitment flow and group selection criteria whereas Table 1 summarizes health assessments per participant group.

**Table 1:** Characteristics of the participants.

	Current swimmers	Past swimmers	Non-swimmers	*p*
**N (males)**	205 (99)	228 (115)	342 (175)	**- -**
**Age** (years, mean ± sd)	8.7 ±0.8	8.6 ±0.7	8.9 ±0.8	**0.011**^¥^
**Weight** (kg)	30.9 (26.6 to 36.9)	32.1 (28.2 to 37.8)	30.8 (26.9 to 37.3)	**0.048**
**Height** (cm)	135 (130 to 139)	136 (131 to 141)	136 (131 to 141)	0.196
**Sport practisers** (%)	100.0	98.3	98.2	0.965
**Lung function**				
FVC (L)	1.88 (1.71 to 2.15)	1.91 (1.71 to 2.18)	1.88 (1.66 to 2.10)	0.125
FEV_1_ (L)	1.73 (1.58 to 1.95)	1.78 (1.60 to 1.99)	1.74 (1.55 to 1.92)	0.081
FEV_1_/FVC (%)	92.8 (89.1 to 96.1)	92.5 (89.0 to 96.6)	92.7 (88.8 to 96.4)	0.998
FEF_25-75_ (L/s)	2.23 (1.97 to 2.71)	2.36 (1.93 to 2.71)	2.27 (1.91 to 2.59)	0.423
PEF (L/s)	3.77 (3.30 to 4.38)	3.77 (3.38 to 4.21)	3.69 (3.27 to 4.27)	0.641
**FEV**_**1**_ **reversibility** (mL)	70 (20 to 130)	60 (10 to 120)	60 (-10 to 110)	**0.028**
**FEV**_**1**_ **reversibility** (%)	4.0 (1.3 to 7.2)	3.4 (0.6 to 6.7)	3.1 (-0.7 to 6.2)	**0.040**
**FVC reversibility** (mL)	40 (-30 to 100)	30 (-30 to 80)	20 (-40 to 90)	0.219
**Exhaled NO** (ppb)	12 (7 to 20)	11 (6 to 20)	10 (5 to 19)	0.086
**Asthma**^β^				
Clinical criteria (n, %)	11.7%	8.3%	9.4%	0.459*
Functional criteria (n, %)	6.3%	7.0%	6.4%	0.957*
Treated asthma (n, %)	6.3%	4.0%	6.1%	0.441*
Ever asthma (n, %)	7.3%	4.8%	7.3%	0.438*
**Atopic eczema** (n, %)	66.7%	62.9%	54.4%	0.465*
**Allergic rhinitis** (n, %)	33.3%	33.8%	31.1%	0.907*
**Allergic sensitization** (%)	32.8	39.5	34.2	0.302*
**Otitis** (n, %)	27.0%	21.0%	32.4%	0.043*
**Pupillometry**				
Maximum (mm, mean ± sd)	5.2 ±0.9	5.3 ±1.0	5.3 ±0.8	0.278^¥^
Minimum (mm, mean ± sd)	3.4 ±0.6	3.4 ± 0.6	3.4 ±0.6	0.537^¥^
CON (%, mean ± sd)	35 ±5	36 ±5	36 ±5	0.203^¥^
ACV (mm/s, mean ± sd)	3.8 ±0.7	3.9 ±0.8	4.0 ±0.7	**0.030**^¥^
MCV (mm/s, mean ± sd)	5.1 ±1.0	5.3 ±1.0	5.4 ±0.9	**0.010**^¥^
ADV (mm/s, mean ± sd)	1.2 ±0.3	1.2 ±0.4	1.2 ±0.3	0.709^¥^
T75 (s, mean ± sd)	1.7 ±0.7	1.7 ±0.7	1.7 ±0.7	0.987^¥^

Data reported as median (P25 to P75) unless otherwise stated. BMI: body mass index; FEV_1_: forced expiratory volume in the first second of FVC; PEF: Peek expiratory flow; FVC: forced vital capacity; FEF_25-75_: forced expiratory flow middle portion of FVC; EBC: exhaled breath condensate; CON: percentage of pupil constriction; ACV: average constriction velocity; MCV: maximum conscription velocity; ADV: average dilation velocity. The p values signalling differences between the three groups were calculated using the Kruskal-Wallis test for non-parametric variables, with the exception of cases marked with (*) which were calculated using qui-square tests, and (^¥^), which were calculated using one-way ANOVA (for normal distributions).

^β^The following operational asthma definitions were adopted: i) Clinical criteria–at least a 12% increase in FEV1 after bronchodilation and over 200mL and/or asthma diagnosed by a physician with reported symptoms (wheezing, dyspnoea or dry cough) occurring in the past 12 months; ii) Functional criteria–at least a 12% increase in FEV1 after bronchodilation and over 200mL; iii) Treated asthma criteria–asthma diagnosed by a physician and currently under inhaled corticosteroid treatment; and iv) Ever asthma–asthma diagnosed by a physician.

### Statistical analysis

The SPSS® statistical package software v20.0 (IBM, USA) was used to statistically analyse the data. The Kolmogorov-Smirnov test was used to check continuous variables for normality. Whenever non-Gaussians distributions were observed, non-parametric tests were used for inferential analysis.

T-student test and one-way ANOVA (for normal distributions) or Mann-Whitney and Kruskal-Wallis tests (for non-parametric distributions) were used to compare continuous variables between two or more than two groups of individuals, respectively. Significant differences were reported with an α-value inferior to 5% (*p*<0.05).

The Spearman’s correlation test was used to find correlations between the number of years in swimming practice and the measurable outcomes. Risk analysis was performed to identify the risk of allergic disease development associated with early swimming and logistic regression analysis was then used to find associations between the number of years in swimming practice and the binary categorical variables. Results were expressed in OR[95%CI] and β[95%CI], respectively. Finally, multinomial logistic regression was used to compare ranked terciles of years in swimming practice to further evaluate the influence of cumulative swimming pool attendance. The results were reported as OR[95%CI].

## Results

**Fig 2:**
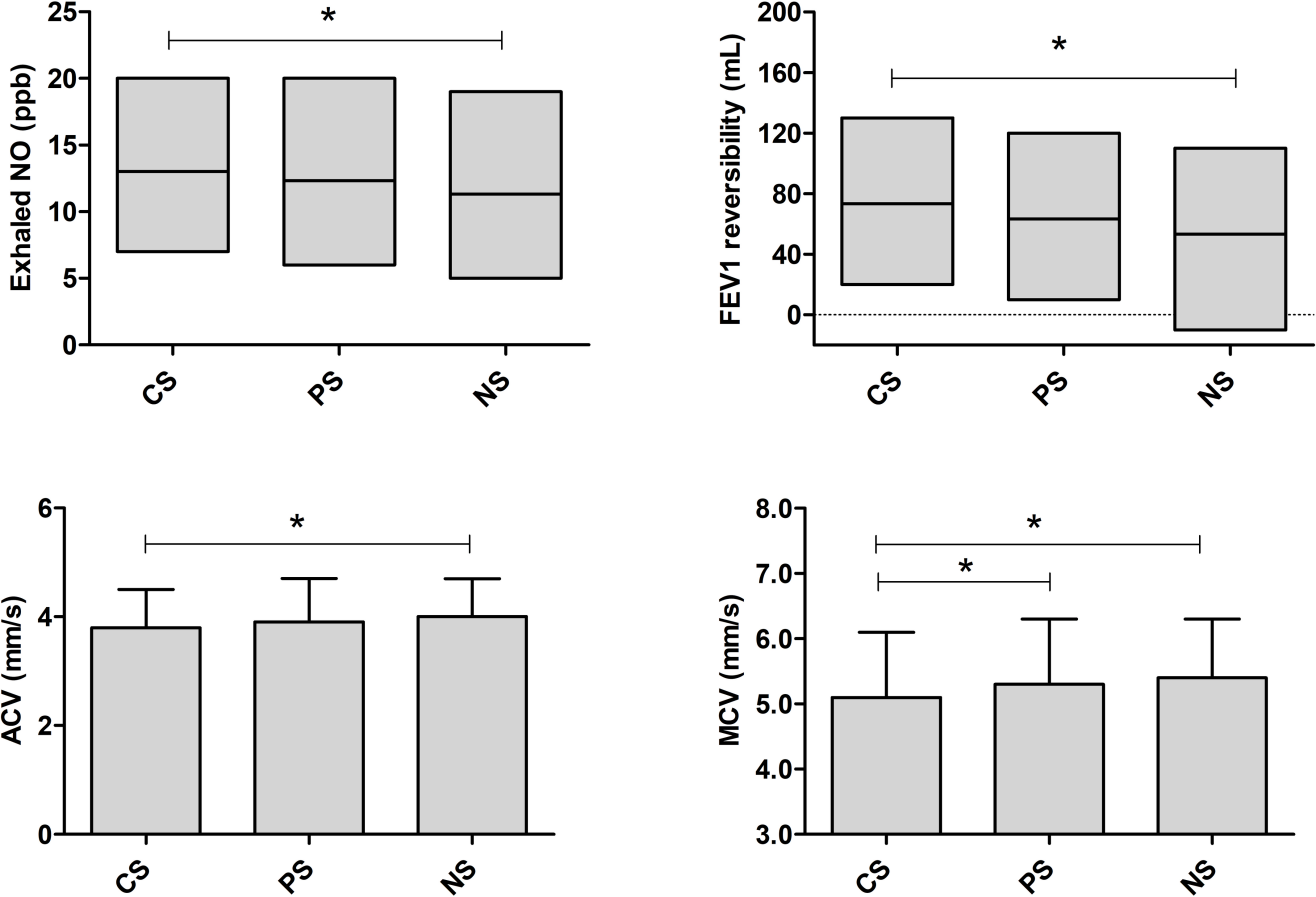
Median (with 25 and 75 percentiles) levels of exhaled NO and FEV1 reversibility, and mean ±SD of measured average constriction velocity (ACV) and maximum constriction velocity (MCV) among the three groups. CS–current swimmers; PS–past swimmers; NS–non-swimmers. *Represents significant differences between two groups indicated by the extremities of the horizontal line (*p*<0.05).

Children in the CS group had significantly lower maximum (MCV) and average pupil constriction velocities (ACV) when compared to both PS and NS groups, MCV (mm/s, mean±sd): 5.1±1.0 *vs* 5.3 ±1.0 *vs* 5.4 ±0.9, respectively (*p* = 0.010); and ACV(mm/s, mean±sd): 3.8 ±0.7 *vs* 3.9 ±0.8 *vs* 4.0 ±0.7, respectively (*p* = 0.030). Moreover, levels of exhaled NO and changes in airway reversibility volume after administration of a beta-2 agonist were significantly higher in the CS group when compared to NS (respectively, median [P25 to P75]: 12ppb [7 to 20] *vs* 10 ppb [5 to 19], *p* = 0.030; and 70mL [20 to 130] vs 60mL [-10 to 110], *p* = 0.007) (Table 1 and Fig 2). These results were not influenced by sedentarism and/or other sport practice since no significant difference was found between percentage of weekly sport practisers between groups (*p* = 0.965).

There were no differences between the three groups of participants for the prevalence of asthma (for any of the 4 definitions) or allergic sensitization, although a significantly higher occurrence of otitis was observed in NS when compared to PS (32.4 *vs* 21.0%, respectively; *p* = 0.013), but not to CS (32.4 *vs* 27.0%, respectively; *p* = 0.251).

Differences found in the studied physiological outcomes were not influenced by the inclusion of individuals with asthma in the groups since, with the exception of atopic eczema, there were no significant changes when exclusively comparing individuals with clinical defined asthma (S1 Table).

As expected, the number of years in swimming practice was significantly higher in CS when compared to PS (mean (±SD) = 3.9 (±2.2) vs 2.4 (±1.6), respectively; *p*<0.010). To investigate the effect of cumulative exposure resultant from swimming practice, correlations between the number of years in swimming practice and continuous variables of the measured health outcomes were calculated using the Spearman’s correlation test (Table 2). Significant correlations were observed for baseline FEV_1_ (rho = 0.11), PEF (rho = 0.18), and the CON (rho = 0.12), ADV (rho = -0.13) and T75 (rho = 0.19) parameters of pupillometry.

**Table 2:** Spearman’s correlation test between continuous clinical parameters and the number of years in swimming practice. Values represent the Spearman’s correlation coefficient. Significant correlations are expressed in bold.

	Number of years in swimming practice (rho)
**Lung function parameters**	
FVC	.098
FEV_1_	**.111**
FEV1/FVC	-.001
FEF 25–75%	.054
PEF	**.184**
Forced expiratory flow	.008
FEV1 reversibility	.064
**Exhaled NO**	.016
**Pupillometry parameters**	
Maximum diameter	.014
Minimum diameter	-.030
CON	**.118**
ACV	.013
MCV	-.001
ADV	**-.126**
T75	**.186**

**Fig 3:**
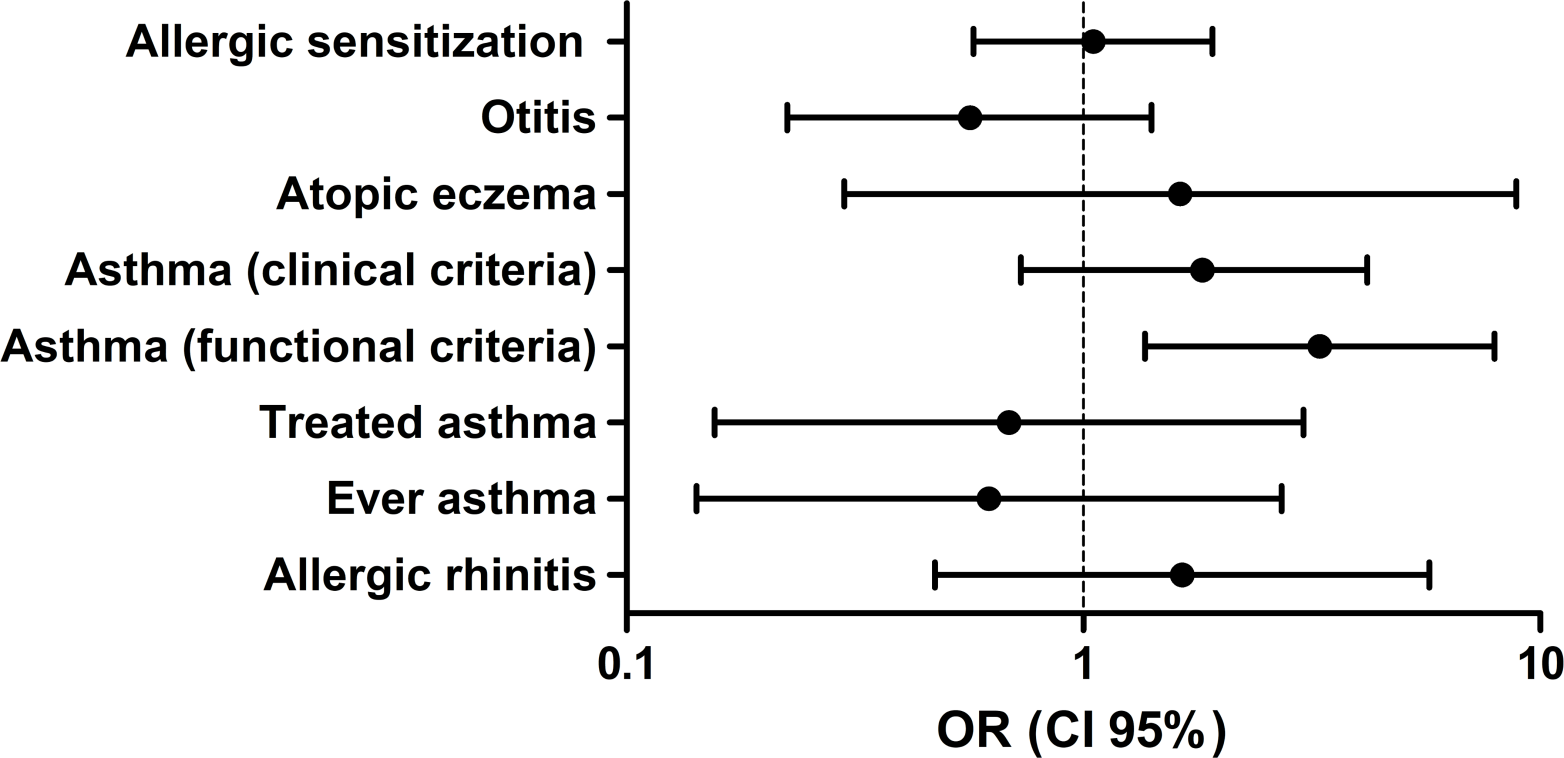
Risk analysis of early swimming (children who started swimming before the age of 3) in the development of allergic diseases and asthma. Results are represented as OR (5–95%CI).

**Fig 4:**
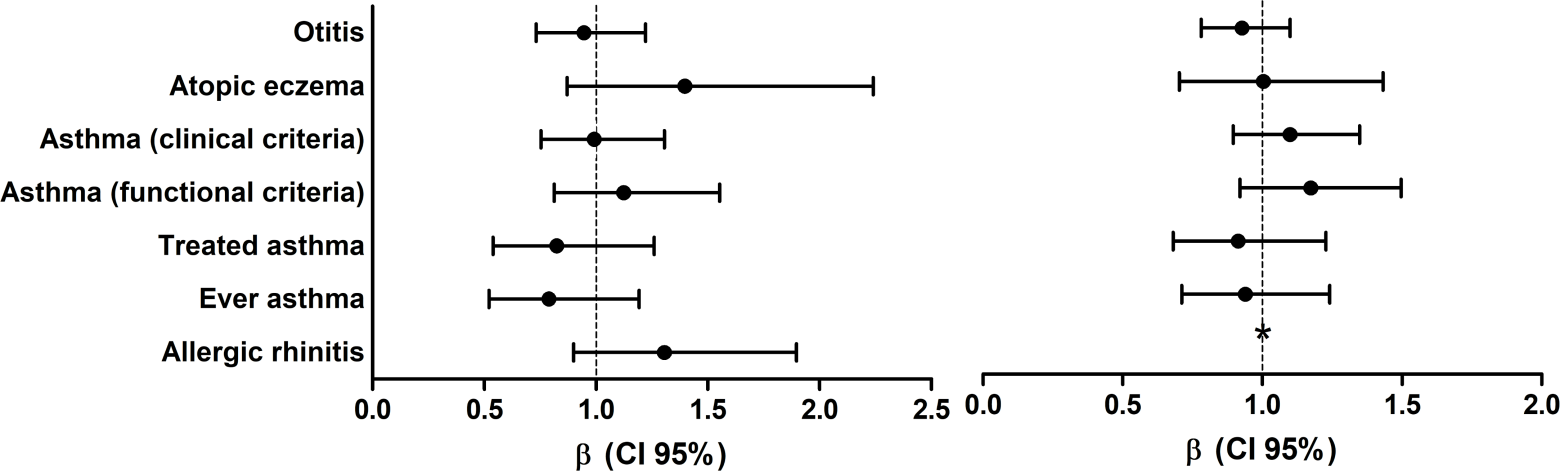
Logistic regression between the number of years in swimming practice and the assessed clinical outcomes (adjusted for age and “early swimming”). Participants were stratified according to their atopic status: A) Atopic children; and B) Non-atopic children. Results are represented as β (5–95%CI). *There were no non-atopic children reporting symptoms of allergic rhinitis.

Early swimming, which was defined as individuals who started swimming before the age of 3, based on a previous study design [29], was associated with a higher tendency for allergic disease development, with a significantly higher risk of asthma defined by the functional criterion (Fig 3). Furthermore, the logistic regression analysis showed a non-significant trend for a higher risk of asthma by lung function criteria (β[95%CI] = Atopic: 1.12[0.81 to 1.55]; Non-atopic: 1.17[0.92 to 1.55]), atopic eczema (β[95%CI] = Atopic: 1.40[0.87 to 2.24]; Non-atopic: 1.00[0.70 to 1.43]) and allergic rhinitis (β[95%CI] = Atopic: 1.31[0.90 to 1.90]) with more years of swimming practice (Fig 4). These results were replicated by the multinomial logistic regression, where a higher risk of asthma (functional criteria), allergic rhinitis and atopic eczema were observed within the highest tercile of years in swimming practice (S1 and S2 Tables).

## Discussion

Our findings provide support to the hypothesis that swimming pool attendance is associated with autonomic changes even in non-elite swimmers. Firstly, we observed by pupillometry that swimming pool exposure in school-aged children is associated with parasympathetic dysautonomia. Secondly, a higher volume of airway reversibility in response to an inhaled beta-2 agonist, suggestive of increased baseline airway smooth muscle constriction, was also observed in children that frequently attend swimming pools. Thirdly, these subtle changes appear to be reversible with swimming practice cessation, as no evidence of parasympathetic perturbances or airway constriction were found in children that used to swim in the past, even if they kept practicing any other type of sport.

As with all proof of concept studies, this one has its limitations. The cross-sectional nature does not allow causal relationships to be established. Nevertheless, the inclusion of a group of participants that were past swimmers may allow the estimation of persistence changes associated with indoor swimming exposure. Another limitation may be inherent to the question used to allocate participants to each group of swimming exposure, since although being “yes or no” questions, they are not exempt of reporting bias. Regarding cumulative exposure, no data on number of hours of training per week has been collected. However, considering the age range of participants, rarely should the practice represent more than 2 hours per week of active swimming. Although continuous outdoor swimming practice is not commonly performed by Portuguese children outside the summer season, information regarding the swimming pool environment has not been collected which may be seen as a limitation. Moreover, knowing the family atopic background could also help to determine if some individuals were already pre-disposed to develop some of these changes. We also cannot exclude reverse causation bias as the number of children with otitis was lower in past swimmers. Nevertheless, our study has several unique strengths. It is community based, not affected by a swimming pool recruitment strategy bias. Moreover, the exclusion of sedentarism-based bias, coupled to the high number of participants per group, as well as the extensive workup evaluation, particularly for assessing the autonomic nervous system and airway physiology, increases the robustness of the findings. Lastly, pupillometry is a sensible method that allows the detection of subtle changes in the oculosympathetic pathways, as previously demonstrated by Yoo *et al* (2017) with the Horner’s eye syndrome [30].

Neurogenic inflammation has been suggested to contribute to the recognized higher prevalence of asthma in athletes training and competing in environments with a high airway irritation potential [31]. Transient receptor potential vanilloid 1 is the centre of almost all neuronal inflammatory signalling pathways; this ion-channel is often co-localized with sensory neuropeptides in the same axon of a primary neuron and its stimulation can lead to the release of these substances. It is expressed in primary sensory neurons, pulmonary smooth muscle cells, bronchial and tracheal epithelial cells and dendritic cells in the lung [22]. Known physical activators of these channels include noxious temperature such as heat or cold, changes in membrane potential, mechanical or osmotic stress, and arachidonic acid metabolites [32]. A recent study showed increased levels of substance P in sputum of competitive swimmers suggesting it may be the result of a compensatory response to the sympathetic stimulation promoted by intensive training, neurogenic inflammatory response to swim stress and/or a local airway chemosensory reflex to chlorine by-products exposure in swimmers [33]. However, altered parasympathetic tonus in healthy swimmers has until this moment been almost exclusively associated with endurance training. Although the high training volumes may certainly influence the autonomic nervous function, this study results now show that parasympathetic dysfunction does occur in healthy children swimmers not undergoing endurance training. Our findings further extend this observation suggesting that autonomic changes may not only be caused by high training volumes, but also by environmental exposure, even in young children. The significantly higher exhaled NO levels observed in swimmers, although subtle and within physiologic reference values, support the co-existence of airways inflammation in those children. Therefore, it is possible that swimming pool exposure may be responsible for airway constriction at two levels: directly, by inhalation of disinfection by-products which will damage airway epithelium and increase oxidative stress [5]; and indirectly, by causing parasympathetic dysautonomia which may lead to a reflex vasoconstriction of bronchial venules, reducing the size of the bronchial lumen and generating increased airways resistance [34].

In the present study, the subtle changes associated with swimming pool attendance tended to disappear in children who discontinued swimming practice, since autonomic parameters, lung function and exhaled NO levels were not significantly different between past and non-swimmers. These results, coupled to the absence of significant correlations between the number of years in swimming practice and the airway reversibility or exhaled NO, suggests that swimming pool environment was the main responsible for the changes in airway inflammation biomarkers, which tend to disappear after ceasing the practice. In addition, parasympathetic activity also appears to be re-established after swimming cessation, since only sympathetic parameters seem to be significantly correlated with the number of years in swimming practice. Although this changes in sympathetic activity may be associated with more years of regular physical exercise [35], this cannot be concluded in the present study since only swimming practice was considered and children may have performed other physical activities at the same time period. Interestingly, these “reversibility” results support the hypothesis presented by Lomax (2016) regarding airway dysfunction in elite swimmers [36]. By systematically reviewing relevant publications, Lomax hypothesised that chlorine exposure in swimming pools coupled to endurance swimming exercises caused epithelial damage that could lead to several airway symptoms, including bronchoconstriction [37], but airway epithelium was estimated to be replenished every 30 to 50 days in the absence of continued damage [36, 38]. When under continuous exposure, the injury-repair process of the airways epithelium may lead to respiratory disorders and, eventually, airway hyperresponsiveness [36, 37, 39]. While children in the present study were certainly not submitted to endurance training, they were still exposed to chlorine-based disinfection by-products.

Although not necessarily a novelty, results also showed that children that started the swimming practice before the age of 3 were tendentially under a higher risk for asthma and allergic disease development, thus supporting the hypothesis presented by Voisin and co-workers in 2014 [29]. This suggests that exposure to a highly chlorinated environment during the early years of life may be even more prejudicial, which further underlines the importance of the practisers’ susceptibility. Nevertheless, and as observed for otitis, reverse-causality bias cannot be excluded and results should be interpreted with caution.

It is important to notice that several positive traits are associated with swimming pool attendance. In line with other population-based studies [1, 40–43], the results showed that swimming pool attendance was not associated with increased of allergic sensitization in children, although a trend for a higher risk of asthma (functional criteria), atopic eczema and rhinitis, was observed in swimmers. Swimming practice was also not associated with a higher prevalence of allergic sensitization and no adverse effects on baseline lung function parameters were observed. In fact, this study shows that children with more years in swimming practice generally have improved baseline lung function, supporting several other studies in the last two decades [44, 45], including those focused in prepubertal children, such as the one published by Courteix *et al* in 1997 [46]. However, seldom has exercise-induced bronchoconstriction been scrutinized in these studies and, as observable in the present study, children that attend swimming pools have a significantly higher exhaled NO and reversibility of FEV_1_. These results, coupled to the altered parasympathetic function in the current swimmers group suggests that exposure during indoor swimming may contribute to airways constriction independently of the training volume and asthma or atopic status.

Two important aspects of the present study need to be taken into consideration: first, the airway reversibility and eosinophilic airway inflammation observed in active swimmers group are non-pathological according to current guidelines [24, 27]; and second, the measured parameters appear to normalize after the practice cessation, as observed with the children in the PS group, independently of continuing other sportive activities. While the physiological mechanisms of the association between altered autonomic function and environmental exposure have not been fully explained, there is evidence showing that traffic-related air pollution may be responsible for disturbances of the autonomic system [47]. Baja *et al* (2013), using structural equation models, also observed that traffic pollution may decrease parasympathetic tone among diabetic elderly [48]. Therefore, we may assume the observed autonomic changes could be associated with indoor swimming pool attendance in susceptible individuals, such as schoolchildren.

## Conclusion

Concluding, swimming pool attendance appears to be associated with autonomic changes even in non-elite swimmers, such as children. Continued swimming pool exposure in school-aged children may cause parasympathetic dysautonomia and increased response to inhaled beta-2 agonists, consequently resulting in increased baseline airway smooth muscle constriction. Although these subtle changes appear to be reversible with swimming practice cessation, it is interesting to note they seem to mirror, in a lower scale, those that characterise swimmers’ asthma: parasympathetic dysautonomia and bronchoconstriction with a neurogenic inflammation component related to high ventilation rates.

## Supporting information

S1 Table Clinical parameters of individuals with asthma, between the three groups. (DOCX)

S2 Table Crude risk analysis between the terciles of years in swimming practice and the development of allergic diseases and asthma. (DOCX)

S3 Table Adjusted risk analysis between the terciles of years in swimming practice and the development of allergic diseases and asthma. (DOCX)

S1 Dataset Database containing the relevant data variables for the study analysis. (SAV)
