# Supplementary material for: Swimming pool exposure is associated with autonomic changes and increased airway reactivity to a beta-2 agonist in school aged children: A cross-sectional survey
Source: PLoS One. 2018 Mar 12;13(3):e0193848. doi: 10.1371/journal.pone.0193848 (PMC5846785; doi:10.1371/journal.pone.0193848)
Supplement: S1 Table — (DOCX) [file pone.0193848.s001.docx]

Table S1. Clinical parameters of individuals with asthma, between the three groups. For this analysis, participants with asthma were selected according to the clinical criteria: at least a 12% increase in FEV1 after bronchodilation and over 200mL and/or asthma diagnosed by a physician with reported symptoms (wheezing, dyspnoea or dry cough) occurring in the past 12 months.

|  | Current swimmers | Past swimmers | Non-swimmers | *p* |
| --- | --- | --- | --- | --- |
| **N (males)** | 24 (10) | 19 (8) | 23 (9) | **- -** |
| **Age** (years, mean ± sd) | 8.9 ±1 | 8.7 ±0.7 | 8.8 ±0.8 | 0.640^¥^ |
| **Weight** (kg) | 33.1 (31.0 to 37.1) | 32.3 (28.9 to 37.5) | 30.7 (26.6 to 39.5) | 0.514 |
| **Height** (cm) | 136 (133 to 140) | 135 (132 to 141) | 135 (129 to 138) | 0.587 |
| **Sport practisers** (%) | 100.0 | 100.0 | 96.9 | 0.506 |
| **Allergic sensitization** (%) | 45.8 | 38.9 | 46.9 | 0.852* |
| **Lung function** |  |  |  |  |
| FEV_1_ (L) | 1.69 (1.50 to 1.95) | 1.72 (1.53 to 1.89) | 1.64 (1.38 to 1.81) | 0.608 |
| PEF (L/s) | 3.54 (3.27 to 4.38) | 3.62 (3.17 to 3.77) | 3.66 (2.86 to 3.93) | 0.667 |
| FVC (L) | 1.87 (1.67 to 2.07) | 1.95 (1.69 to 2.08) | 1.86 (1.58 to 2.05) | 0.473 |
| FEF_25-75_ (L/s) | 2.13 (1.67 to 2.79) | 1.90 (1.62 to 2.62) | 2.01 (1.63 to 2.59) | 0.535 |
| FEV_1_/FVC (%) | 92.8 (87.6 to 96.0) | 87.1 (83.5 to 94.1) | 92.2 (85.3 to 96.9) | 0.283 |
| **FEV_1_ reversibility** (mL) | 22 (5 to 27) | 23 (21 to 33) | 22 (11 to 30) | 0.388 |
| **FVC reversibility** (mL) | 11 (3 to 23) | 19 (3 to 28) | 18 (2 to 24) | 0.636 |
| **Exhaled NO** (ppb) | 20 (12 to 45) | 15 (8 to 28) | 20 (4 to 42) | 0.572 |
| **Otitis** (n, %) | 35.3% | 26.7% | 30.8% | 0.870* |
| **Atopic eczema** (n, %) | 100% | 83.3% | 33.3% | **0.048*** |
| **Allergic rhinitis** (n, %) | 41.7% | 50.0% | 45.8% | 0.916* |
| **Pupillometry** |  |  |  |  |
| Maximum (mm, mean ± sd) | 5.2 ±1.1 | 5.3 ±0.8 | 5.3 ±0.7 | 0.908^¥^ |
| Minimum (mm, mean ± sd) | 3.4 ±0.9 | 3.4 ± 0.5 | 3.4 ±0.5 | 0.932^¥^ |
| CON (%, mean ± sd) | 35 ±5 | 36 ±5 | 35 ±5 | 0.982^¥^ |
| ACV (mm/s, mean ± sd) | 3.6 ±0.8 | 3.9 ±0.7 | 4.0 ±0.7 | 0.166^¥^ |
| MCV (mm/s, mean ± sd) | 5.0 ±1.1 | 5.5 ±1.0 | 5.3 ±1.0 | 0.324^¥^ |
| ADV (mm/s, mean ± sd) | 1.2 ±0.3 | 1.0 ±0.2 | 1.1 ±0.3 | 0.146^¥^ |
| T75 (s, mean ± sd) | 2.0 ±0.7 | 1.7 ±0.9 | 1.7 ±0.6 | 0.444^¥^ |

Data reported as median (P25-P75) unless otherwise stated. BMI: body mass index; FEV_1_: forced expiratory volume in the first second of FVC; PEF: Peek expiratory flow; FVC: forced vital capacity; FEF_25-75_: forced expiratory flow middle portion of FVC; EBC: exhaled breath condensate; CON: percentage of pupil constriction; ACV: average constriction velocity; MCV: maximum conscription velocity; ADV: average dilation velocity. The p values signalling differences between the three groups were calculated using the Kruskal-Wallis test for non-parametric variables, with the exception of cases marked with (*) which were calculated using qui-square tests, and (^¥^), which were calculated using one-way ANOVA (for normal distributions).
