# Supplementary material for: Swimming pool exposure is associated with autonomic changes and increased airway reactivity to a beta-2 agonist in school aged children: A cross-sectional survey
Source: PLoS One. 2018 Mar 12;13(3):e0193848. doi: 10.1371/journal.pone.0193848 (PMC5846785; doi:10.1371/journal.pone.0193848)
Supplement: S2 Table — (DOCX) [file pone.0193848.s002.docx]

| **Crude** | | | | | |
| --- | --- | --- | --- | --- | --- |
| **Variable** | **Tercile of years in swimming practice** | **OR** | **CI 95%**  **Lower bound** | **CI 95%**  **Upper bound** | ***p*** |
| Asthma (clinical criteria) | 1 (Reference) | 1 | -- | -- | -- |
|  | 2 | 0.661 | 0.288 | 1.514 | 0.327 |
|  | 3 | 0.991 | 0.458 | 2.146 | 0.982 |
| Asthma (functional criteria) | 1 (Reference) | 1 | -- | -- | -- |
|  | 2 | 0.641 | 0.216 | 1.899 | 0.422 |
|  | 3 | 1.427 | 0.563 | 3.615 | 0.454 |
| Treated asthma | 1 (Reference) | 1 | -- | -- | -- |
|  | 2 | 1.132 | 0.409 | 3.134 | 0.811 |
|  | 3 | 0.648 | 0.200 | 2.098 | 0.470 |
| Ever asthma | 1 (Reference) | 1 | -- | -- | -- |
|  | 2 | 0.990 | 0.370 | 2.648 | 0.983 |
|  | 3 | 0.680 | 0.229 | 2.018 | 0.487 |
| Allergic rhinitis | 1 (Reference) | 1 | -- | -- | -- |
|  | 2 | 0.933 | 0.359 | 2.428 | 0.888 |
|  | 3 | 1.527 | 0.567 | 4.114 | 0.402 |
| Otitis | 1 (Reference) | 1 | -- | -- | -- |
|  | 2 | 1.102 | 0.579 | 2.097 | 0.767 |
|  | 3 | 0.655 | 0.326 | 1.316 | 0.235 |
| Atopic eczema | 1 (Reference) | 1 | -- | -- | -- |
|  | 2 | 1.200 | 0.281 | 5.124 | 0.806 |
|  | 3 | 1.325 | 0.351 | 5.000 | 0.678 |

**S2 Table. Crude risk analysis between the terciles of years in swimming practice and the development of allergic diseases and asthma.** Tercile 1 represents the lowest exposure, while tercile 3 represents the highest. Results are expressed as odds ratio with 95% confidence interval.
