# Supplementary material for: Swimming pool exposure is associated with autonomic changes and increased airway reactivity to a beta-2 agonist in school aged children: A cross-sectional survey
Source: PLoS One. 2018 Mar 12;13(3):e0193848. doi: 10.1371/journal.pone.0193848 (PMC5846785; doi:10.1371/journal.pone.0193848)
Supplement: S3 Table — (DOCX) [file pone.0193848.s003.docx]

| **Adjusted for gender and atopic status** | | | | | |
| --- | --- | --- | --- | --- | --- |
| **Variable** | **Tercile of years in swimming practice** | **β** | **CI 95%**  **Lower bound** | **CI 95%**  **Upper bound** | ***p*** |
| Asthma (clinical criteria) | 1 (Reference) | 1 | -- | -- | -- |
|  | 2 | 0,594 | 0,253 | 1,395 | 0,232 |
|  | 3 | 1,108 | 0,507 | 2,422 | 0,796 |
| Asthma (functional criteria) | 1 (Reference) | 1 | -- | -- | -- |
|  | 2 | 0,536 | 0,170 | 1,686 | 0,286 |
|  | 3 | 1,551 | 0,607 | 3,964 | 0,359 |
| Treated asthma | 1 (Reference) | 1 | -- | -- | -- |
|  | 2 | 1,118 | 0,402 | 3,110 | 0,830 |
|  | 3 | 0,726 | 0,222 | 2,371 | 0,596 |
| Ever asthma | 1 (Reference) | 1 | -- | -- | -- |
|  | 2 | 0,973 | 0,362 | 2,617 | 0,958 |
|  | 3 | 0,753 | 0,251 | 2,255 | 0,612 |
| Allergic rhinitis | 1 (Reference) | 1 | -- | -- | -- |
|  | 2 | 1,662 | 0,306 | 9,029 | 0,557 |
|  | 3 | 5,212 | 0,714 | 38,054 | 0,104 |
| Otitis | 1 (Reference) | 1 | -- | -- | -- |
|  | 2 | 1,175 | 0,611 | 2,261 | 0,629 |
|  | 3 | 0,623 | 0,304 | 1,276 | 0,196 |
| Atopic eczema | 1 (Reference) | 1 | -- | -- | -- |
|  | 2 | 1,385 | 0,305 | 6,284 | 0,673 |
|  | 3 | 1,240 | 0,319 | 4,819 | 0,756 |

**S3 Table. Adjusted risk analysis between the terciles of years in swimming practice and the development of allergic diseases and asthma.** Tercile 1 represents the lowest exposure, while tercile 3 represents the highest. Results are expressed as odds ratio with 95% confidence interval. The model was adjusted for gender and atopic status (allergic sensitization in this study).
